# The Influence of Dyslexia Candidate Genes on Reading Skill in Old Age

**DOI:** 10.1007/s10519-018-9913-3

**Published:** 2018-06-29

**Authors:** Michelle Luciano, Alan J. Gow, Alison Pattie, Timothy C. Bates, Ian J. Deary

**Affiliations:** 1 Centre for Cognitive Ageing and Cognitive Epidemiology, University of Edinburgh, Edinburgh, Scotland UK; 2 Department of Psychology, University of Edinburgh, 7 George Square, Edinburgh, EH9 8JZ Scotland UK; 3 Department of Psychology, Heriot Watt University, Edinburgh, EH14 4AS Scotland, UK

**Keywords:** Dyslexia, Neuronal migration, Axon guidance, Reading ability, Lothian birth cohort

## Abstract

A number of candidate genes for reading and language impairment have been replicated, primarily in samples of children with developmental disability or delay, although these genes are also supported in adolescent population samples. The present study used a systematic approach to test 14 of these candidate genes for association with reading assessed in late adulthood (two cohorts with mean ages of 70 and 79 years). Gene-sets (14 candidates, axon-guidance and neuron migration pathways) and individual SNPs within each gene of interest were tested for association using imputed data referenced to the 1000 genomes European panel. Using the results from the genome-wide association (GWA) meta-analysis of the two cohorts (N = 1217), a competitive gene-set analysis showed that the candidate gene-set was associated with the reading index (p = .016) at a family wise error rate corrected significance level. Neither axon guidance nor neuron migration pathways were significant. Whereas individual SNP associations within *CYP19A1, DYX1C1, CNTNAP2* and *DIP2A* genes (p < .05) did not reach corrected significance their allelic effects were in the same direction as past available reports. These results suggest that reading skill in normal adults shares the same genetic substrate as reading in adolescents, and clinically disordered reading, and highlights the utility of adult samples to increase sample sizes in the genetic study of developmental disorders.

**Electronic supplementary material:**

The online version of this article (10.1007/s10519-018-9913-3) contains supplementary material, which is available to authorized users.

## Introduction

Reading performance is highly heritable (Bates et al. 2004; Hayiou-Thomas et al. 2010), making the genetic study of this skill important for understanding the causes of disorders in these abilities (Fisher and DeFries 2002). A number of candidate genes influencing reading and language impairment have been discovered (e.g., Francks et al. 2004; Meng et al. 2005a, b) and have replicated in population samples of adolescents and young adults (Bates et al. 2010; Luciano et al. 2007; Scerri et al. 2011). However, it is known that larger samples are required to successfully search the genome for complex trait variants (Price et al. 2015) if desired outcomes such as substantive polygenic risk scores (Luciano et al. 2017) and new gene discoveries important for understanding biological pathways (Wang et al. 2010) are to be made. Child samples are, however, typically not on the scale required. One possible route forward in the genetics of reading disorder would be to utilise samples of older adults to facilitate large GWA studies which could inform developmental disorders. A barrier to large-scale investment in this approach is that it is currently unknown whether reading disorder in adults follows the same genetic pattern as in children, or even if genes associated with reading replicate in older adults, with many years of practice at the skill. To validate this approach, here, we tested association between 14 candidate genes—implicated in reading impairment—and reading ability in two elderly cohorts.

There are no longitudinal studies specifically on reading skill spanning childhood past young adulthood, but variation in normal cognitive ability has been shown to be stable over the life course (Deary et al. 2000). Further, measures of reading comprehension taken in high-school have been shown to explain ~ 80% of variance in adult reading, measured 21 or 26 years later (Smith 1993). This suggests that correlated measures of reading skill would be similarly predictive over time. Children with reading impairment can improve with intervention (Foorman et al. 2003), and whereas strategies to compensate for specific weaknesses can develop, reading impairment does not completely disappear in adulthood (Lefly and Pennington 1991). Nonlinear latent growth models showed that a broad reading and writing index had the largest rate of change (faster growth) between ages 2 and 19 years and the second lowest change (slower decline) across the ages 20–75 years compared to other cognitive abilities (McArdle et al. 2002). Variability in acquisition of reading during childhood is influenced by genetic and environmental factors although maximal reading and writing performance is not achieved until the mid-20s (McArdle et al. 2002). Thus, genetic effects which for reading skill increase during development and are mostly stable across time (Hart et al. 2013) might be greatest once this maximal attainment is reached. It may be, then, that the genetic study of reading ability in adults is a more (rather than less) sensitive approach for identifying genes.

Genetic association studies of reading traits have primarily been performed in samples of children, adolescents and young adults, particularly those with dyslexia. A number of candidate genes for reading impairment have been discovered, with successful replications in independent samples (Scerri and Schulte-Koene 2010), including reading measures collected in childhood and adolescent general-population samples (Luciano et al. 2007; Paracchini et al. 2011). We predicted that such candidate genes would show association with reading in older adults. As a starting point we included 11 genes that were recently exon sequenced and systematically tested for association with developmental dyslexia by Matsson and colleagues (2015). Of these, seven—*CYP19A1, DCDC2, DYX1C1, GCFC2* (or *C2orf3*), *KIAA0319, MRPL19*, and *ROBO1*—were found by initial linkage evidence which then led to associations with specific genetic variants that were replicated in multiple independent studies (see reviews by Raskind et al. 2012; Scerri and Schulte-Koene 2010). Table 1 indicates the level of existing support for these candidate genes based on positive and null associations between SNPs in these genes and dyslexia/reading-related measures. The four additional genes—*DIP2A, PRMT2, PCNT*, and *S100B*—located in a region on 21q22.3 were identified through segregation analysis in a Dutch family with developmental dyslexia (Poelmans et al. 2009). Of these, *S100B* has since received independent support in German families as a developmental dyslexia candidate gene (Matsson et al. 2015). Two further genes—*CNTNAP2* and *CMIP*—were part of a dyslexia candidate gene set tested for replication by Carrion-Castillo et al. (2017) because they had been associated with reading measures in at least two independent samples. Finally, *KIAA0319L* has been the focus of dyslexia association studies (Couto et al. 2008) and lies in an independently replicated linkage region (Scerri and Schulte-Koene 2010). We thus examined a total of 14 candidate genes.

To test association, we focussed on individual SNPs within these genes previously tested for association with reading dis(ability) and gene-set analysis which included (1) the 14 candidate genes, (2) genes within the axon guidance pathway, and (3) genes within the neuron migration pathway. The axon guidance (GO:0007411: “chemotaxis process that directs the migration of an axon growth cone to a specific target site”) and neuron migration (GO:0001764: “movement of an immature neuron from germinal zones to specific positions where they will reside as they mature”) pathways are theorised to be prominent biological pathways involved in reading impairment (Poelmans et al. 2011) based on the function of the dyslexia candidate genes.

## Materials and methods

### Participants

#### The Lothian Birth Cohort of 1936 (LBC1936)

This cohort, born in 1936, mostly resided in the Edinburgh region of Scotland and consisted of 1091 community dwelling participants (49.8% women) (Deary et al. 2012, 2004). They were aged approximately 70 years at collection of the cognitive phenotypes used in this study and were relatively healthy at the time of testing; none had dementia. The sample score higher on childhood intelligence and have higher socio-economic status (SES) than the general population (Deary et al. 2012). Blood was obtained by trained nurses to extract DNA for genotyping at the time of their clinical visit. Ethics permission was obtained from the Multi-Centre Research Ethics Committee for Scotland (MREC/01/0/56), the Lothian Research Ethics Committee (LREC/2003/2/29). Written informed consent was given by all participants.

#### The Lothian Birth Cohort of 1921 (LBC1921)

This cohort, born in 1921, mostly resided in the Edinburgh region of Scotland and consist of 550 relatively healthy community dwelling participants (57.4% women) (Deary et al. 2004, 2012). They were aged approximately 79 years at collection of the cognitive phenotypes used in this study; exclusions were made for dementia. Like LBC1936, this sample differed from the general population in childhood intelligence and SES. Blood was obtained by trained nurses to extract DNA for genotyping at the time of their clinical visit. Ethics permission was obtained from the Lothian Research Ethics Committee (LREC/1998/4/183). Written informed consent was given by all participants.

### Genotyping

DNA, extracted from blood samples, was genotyped on the Illumina 610-Quadv1 whole-genome SNP array (Illumina, San Diego, CA, USA) by the Genetics Core Laboratory at the Wellcome Trust Clinical Research Facility, Western General Hospital, Scotland. Standard genotype quality control procedures were performed, including checks for gender discrepancies, individual relatedness, and non-Caucasian ascent (for more details see, Houlihan et al. 2010). Necessary exclusions resulted in a final sample of 1005 individuals. Population stratification was controlled using the first four factors extracted from a Multidimensional scaling analysis (Li and Yu 2007) of the identity-by-state distance matrix, as previously detailed for these cohorts (Davies et al. 2011). These were used as covariates in the genetic association analyses. Imputation to the 1000G European reference panel (phase 1 v3) was done using Minimac (van Leeuwen et al. 2015). Only SNPs with a minor allele frequency greater than 5% and with an imputation quality score (r^2^) greater than .30 were retained for analysis.

### Measures

#### LBC1936

Two word recognition tests—National Adult Reading Test, NART (Nelson and Willison 1991); Wechsler Test of Adult Reading, WTAR (Corporation 2001)—were administered. Both of these tests require the correct pronunciation of low-frequency irregular words and therefore scores on both tests are primarily used as estimates of pre-morbid IQ, that is, they are not indexing the functioning of the lexical storage system per se (Coltheart et al. 2001), but also index vocabulary, which is typically larger in people with higher IQs (Dykiert and Deary 2013). To produce a measure of reading skill independent of IQ, we ran a principal components analysis on a battery of tests: a general cognitive ability test (a modified version of the Moray House Test (MHT) no. 12 (Education 1949)) which examined a variety of mental abilities (e.g., reasoning, arithmetic, spatial, verbal); two nonverbal IQ tests (Wechsler Matrix Reasoning, Wechsler Block Design; (Wechsler 1998)); a phonemic verbal fluency test (the summed score of trials requiring production of words starting with the letters C, F and L; Lezak 2004); a self-reported measure of book reading frequency (on a five-point scale from “every day or about every day” to “less than once a year/never,”), and NART and WTAR. We selected book reading in preference to other types of reading (e.g., newspaper) because in the National Adult Literacy survey of almost 25,000 adults, it was more predictive of proficient prose and document literacy than other forms of reading content (Smith 1996). Based on the scree plot, two components had eigenvalues greater than 1; an orthogonal (varimax) rotation was used to obtain maximal separation of the reading from the general cognitive ability component. The first component, explaining 38% of variance, was the general cognitive ability factor (loadings above 0.73 on MHT, Matrix Reasoning, and Block Design and loadings of 0.54 for the NART and WTAR). The second component explained 30% of variance, and assessed reading independent of IQ. Frequency of book reading loaded highly (0.77) on this component, as did the NART (0.72 loading) and WTAR (0.73), and, to a lesser extent, verbal fluency (0.51) and MHT (0.44).This indicated that reading ability could be assessed independent of general cognitive ability. Component scores for the reading component were calculated using a regression approach for those participants with complete data and with genotyping, giving a sample of 879 (436 male) with a mean age of 69.5 years (SD = 0.84).

A previous magnetic resonance imaging study in this cohort showed that a verbal executive processing factor partly underlies verbal fluency scores and was associated with a major language-related white matter tract (Hoffman et al. 2017). We therefore calculated such an index by obtaining residual scores from the regression of verbal fluency scores on a standardised composite measure of the NART and WTAR (that is, removing word storage variation from the verbal fluency scores to tap executive processing variation related to their access). Missing data resulted in a sample of 1000 (507 male) with genotyping data with a mean age of 69.6 years (SD = 0.84) for this measure. Given that verbal executive processing has not been the focus of previous genetic studies, we treat these analyses as exploratory and their results are shown in the online supporting material.

#### LBC1921

Members of this sample had also completed the NART (but not the WTAR), the verbal fluency test, and MHT. In addition, they had completed the Raven’s Standard Progressive Matrices (Raven et al. 1977), a measure of general nonverbal reasoning ability. Roughly four years later, a self-reported measure of lifetime reading (books, newspaper, and magazines) and writing was gathered from a retrospective questionnaire. The lifetime reading component extracted from this questionnaire was primarily defined by book reading.

To obtain a measure of reading ability independent of general cognitive ability, a principal components analysis of each of these measures was performed using varimax rotation, with two components retained based on their eigenvalues being greater than 1. The first component, explaining 48% of variance, tapped general cognitive ability, loading 0.90 and 0.87 on MHT and Raven’s, respectively. As expected, NART and verbal fluency also loaded substantially on this general component with respective loadings of 0.75 and 0.54, with the rotated component score approach allowing us to control for this g-loaded component of the tests. The second orthogonal component, explaining 24% of shared variance, was defined most strongly by lifetime reading (0.92), NART and verbal fluency (loadings of 0.40), and thus tapped reading skill in this sample. Reading component scores were produced for 365 individuals due to a reduced sample for the lifetime reading component; 338 (138 male) with a mean age of 79.1 years (SD = 0.56) had genetic data. A verbal executive processing index was created from residual scores obtained from the regression of verbal fluency on standardised NART. For this supplementary analysis, scores were available for 503 (208 male) genotyped individuals with a mean age of 79.1 years (SD = 0.6).

### Statistical analyses

Genome-wide SNP association analysis was performed using mach2qtl (Li et al. 2009) under an additive model, and controlling for age, gender, and the first four population stratification components. Analyses were conducted separately in the LBC1936 and LBC1921 cohorts and the results meta-analysed using a weighted inverse variance method in METAL (Willer et al. 2010). Genome-wide SNP association results were used to evaluate the over-representation of significant associations in an omnibus test of the 14 candidate genes as a gene set; follow-up analyses were performed for each individual gene. Further, two biological pathways—neuron migration (containing 103 genes) and axon guidance (containing 203 genes)—were tested for overrepresentation of significant associations. Relevant genes in these pathways (defined by respective GO:0001764 and GO:0007411 terms) were downloaded from the gene ontology database, http://amigo.geneontology.org/amigo. Gene set tests (i.e., candidate gene set, neuron migration, axon guidance) were performed in MAGMA (de Leeuw et al. 2015) using a competitive gene-set approach, which, in essence, tests whether there is greater association of SNPs in the specified gene set compared to other genes. By default, MAGMA conditions the gene set on gene size, gene density (relative level of linkage disequilibrium between SNPs) and the inverse of the mean minimum allele count in the gene (to guard against reduced power for those SNPs with low frequency). SNPs within a gene are defined by the transcription start and stop sites of that gene. Because our gene sets overlapped, Bonferroni correction for multiple testing was deemed too conservative. Instead, in MAGMA, we established a family-wise error rate corrected alpha level (p < .017) empirically using 20,000 permutations—this large number of permutations was needed because the corrected p value was close to the significance level.

The 14 candidate genes contained 9225 SNPs, 62 of which have been previously associated with reading ability/disability (Becker et al. 2014; Carrion-Castillo et al. 2017; Couto et al. 2008; Paracchini et al. 2011; Poelmans et al. 2011), and included variants identified through Fluorescent In Situ Hybridization and SNP microarray analyses of a deletion on 21q22.3 but not specifically tested for association (Table II in, Poelmans et al. 2009). Based on 47.81 independent tests, as determined by the matrix spectral decomposition software which considers marker linkage disequilibrium (Nyholt 2004), replication level support for these single SNPs was judged against a Bonferroni-adjusted p value of .001.

## Results

In LBC1936, (standardised) component scores for the reading component were normally distributed with no extreme scores (range: − 3.5 to 2.2; skew: − .51). Individuals with scores less than 2.5 SD from the mean had Mini-Mental State Examination (Folstein et al. 1975) scores above 25 indicating that they were not affected by undiagnosed dementia. In LBC1921, (standardised) component scores for the reading component were normally distributed with no outliers (range: − 2.5 to 2.1; skew: − .22).

**Fig. 1:**
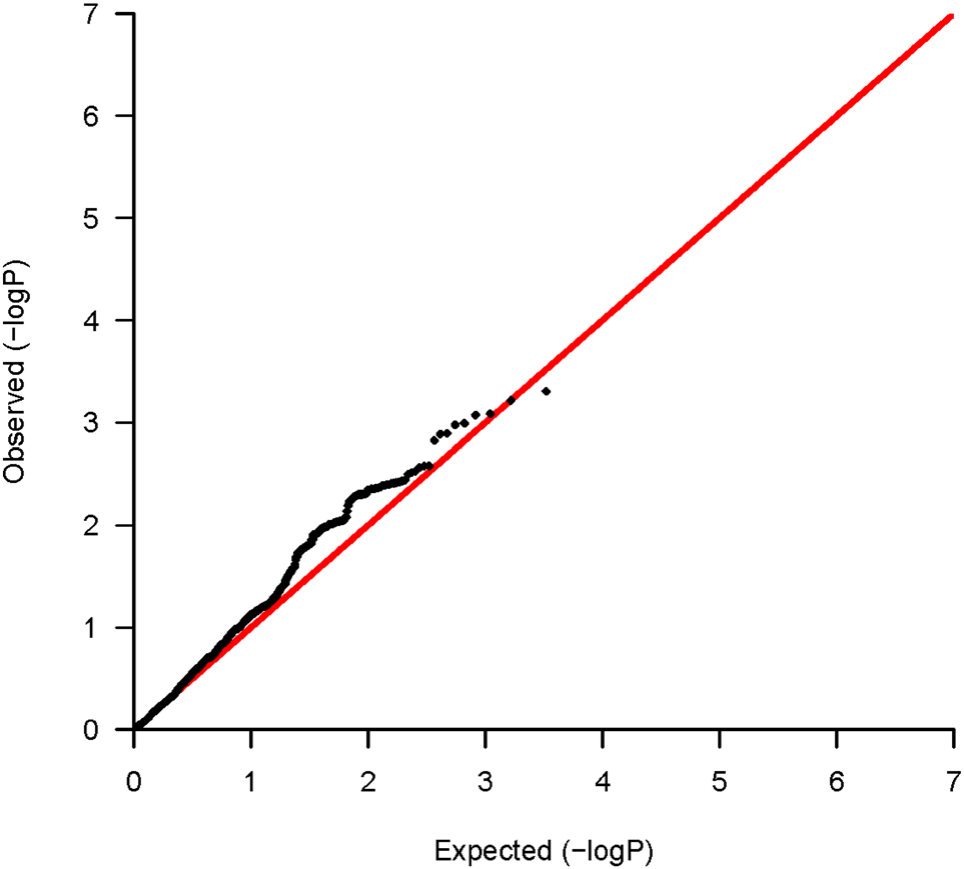
QQ Plot for dyslexia candidate SNP associations (N = 9225 SNPs) with the reading component

The QQ plot for the reading component (Fig. 1) is shown for all SNPs lying within the dyslexia candidate genes of interest; see online Fig. 1 for verbal executive processing. There is some positive deviation in observed p values from the null distribution, attributable to genetic signal. The genome-wide SNP association results (downloadable from http://www.ccace.ed.ac.uk/) were used in the gene-based tests. Analysed as a broad ‘candidate’ gene-set, significant SNPs within the 14 candidate genes were overrepresented among our results for the reading component (Standardised Beta = .014, Beta = 0.51, SE = 0.24, p = .016) but not for verbal executive processing (p > .05). Results for the individual genes within the significant gene set are shown in Table 1. Based on their p-values, it may be that *CYP19A1, DYX1C1, DIP2A*, and *KIAA0319L* contribute more to the overall association between the candidate gene set and reading ability. Gene-set analysis of neuron migration and axon guidance pathways were not significant for the reading component or verbal executive processing (p > .05).

**Table 1:** Gene-based results of 14 reading disability candidate genes tested for association with reading ability in elderly cohorts

Gene	Chr	Start	Stop	N SNPs	N parameters	N	Zstat	p	Previous association studies of SNPs within gene		
									Measures	Association*	Null finding
*KIAA0319L* ^a^	1	35899091	36023037	18	3	1217	1.574	0.058	Reading disability	Couto et al. (2008)	
*MRPL19*	2	75873909	75889334	39	5	1217	− 0.081	0.532	Dyslexia status; reading disability; reading measures in dyslexic/SLI families	Anthoni et al. (2007); Newbury et al. (2011)	Becker et al. (2014); Matsson et al. (2015); Scerri et al. (2011); Venkatesh et al. (2013)
*GCFC2*	2	75889831	75938111	88	8	1217	− 0.214	0.585			
*ROBO1*	3	78646388	79817059	1900	56	1217	− 1.342	0.910	Dyslexia status; mental calculation in dyslexics; reading disability; reading skill	Mascheretti et al. (2014); Sun et al. (2017); Tran et al. (2014)	Matsson et al. (2015); Venkatesh et al. (2013)
*DCDC2*	6	24171983	24383520	580	32	1217	− 0.000	0.500	Dyslexia status; discriminant score; reading skill	Chen et al. (2017); Cope et al. (2012); Deffenbacher et al. (2004); Lind et al. (2010); Matsson et al. (2015); Muller et al. (2016); Newbury et al. (2011); Scerri et al. (2011); Wilcke et al. (2009)	Newbury et al. (2011); Cope et al. (2012); Brkanac et al. (2007)
*KIAA0319*	6	24544332	24646383	231	21	1217	− 0.287	0.613	Single word reading; dyslexia status	Cope et al. (2005a, b); Couto et al. (2010); Darki et al. (2012); Dennis et al. (2009); Harold et al. (2006); Muller et al. (2016); Newbury et al. (2011); Paracchini et al. (2008); Rice et al. (2009); Scerri et al. (2011); Shao et al. (2015); Venkatesh et al. (2013)	Brkanac et al. (2007); Luciano et al. (2007); Schumacher et al. (2006)
*CNTNAP2*	7	1.46E + 08	1.48E + 08	6211	190	1217	0.038	0.485	Nonword repetition; nonword reading fluency (x educational time-point); reading measures (in SLI families)	Carrion-Castillo et al. (2017); Newbury et al. (2011); Vernes et al. (2008); Whitehouse et al. (2011)	
*CYP19A1*	15	51500254	51630795	254	18	1217	1.996	0.023	Dyslexia status; reading skills	Anthoni et al. (2012)	Matsson et al. (2015)
*DYX1C1*	15	55709953	55800432	236	15	1217	2.384	0.009	Dyslexia; orthographic choice	Bates et al. (2010); Brkanac et al. (2007); Dahdouh et al. (2009); Taipale et al. (2003); Wigg et al. (2004); Zhang et al. (2012)	Bellini et al. (2005); Cope et al. (2005a, b); Marino et al. (2005); Meng et al. (2005a, b); Scerri et al. (2004)
*CMIP*	16	81478775	81745367	844	75	1217	1.091	0.138	Spelling; nonword repetition	Newbury et al. (2009); Scerri et al. (2011)	Carrion-Castillo et al. (2017)
*PCNT*	21	47744036	47865682	394	16	1217	1.188	0.117	Dyslexia status	Poelmans et al. (2009)^b^	Matsson et al. (2015)
*DIP2A*	21	47878862	47989926	343	21	1217	2.049	0.020	Dyslexia status	Poelmans et al. (2009)^b^	Matsson et al. (2015)
*S100B*	21	48018531	48025035	27	7	1217	− 0.032	0.513	Dyslexia status	Matsson et al. (2015)	
*PRMT2*	21	48055507	48085155	71	7	1217	1.196	0.116	Dyslexia status	Poelmans et al. (2009)^b^	Matsson et al. (2015)

Based on Human Genome Assembly Build 37

*Includes marginal association and inconsistent direction of allelic effect

^a^Previous association studies tested intergenic SNPs and haplotypes near *MRPL19-GCFC2*

^b^Supported by co-segregation with dyslexia

With regard to individual SNP association we focus on 62 SNPs within the candidate genes previously linked to reading ability/disability. Results for these SNPs are presented in online Table 1 (see online Table 2 for verbal executive processing). None reached the corrected significance level.

## Discussion

The question of whether genetic influences on developmental disorders can be detected in adulthood is an important one because it broadens the sampling frame available to study such traits. Not only did our study replicate genetic effects found for specific reading disorder, but it did so in a sample of older individuals (≥ 70 years) who were not reading impaired. The reading skill index was associated with the 14 candidate gene-set as a whole, with the strongest individual gene support coming from *DYX1C1, DIP2A, CYP19A1 and KIA00319L*. Axon guidance and neuron migration pathways were not significant.

Given that the results of our replication study might be of interest to future meta-analyses in the area, it is important to more closely examine the individual SNP effects despite them attaining only nominal significance. Within *DYX1C1*, now known as Dynein Axonemal Assembly Factor 4 (*DNAAF4*), the direction of allelic effect for rs3743204 (the most significant variant) was consistent with Bates et al. (2010), and Becker et al. (2014) who found the minor allele associated with better reading scores in respective population and reading-impaired samples. Our effect for rs7174102 was in the same direction as Paracchini et al. (2011) who, for a population-based sample, reported a negative effect of the minor allele on spelling scores. And in this same sample, reading scores were positively associated with the minor allele of rs8040756, consistent with our findings. For *CNTNAP2*, rs759178, rs17236239 and rs2710102 have been previously associated with language traits in the general population (Luciano et al. 2013; Whitehouse et al. 2011) and in impairment (also rs4431523, Vernes et al. 2008); our direction of allelic effect for these SNPs was consistent with these studies. Note that Carrion-Castillo et al. (2017) reported the opposite direction of effect for rs17236239. None of the nominally significant SNPs in *DIP2A* have been previously reported in association studies of reading impairment/ability and here we show that they might have effects on verbal executive processing as well as reading ability. The only other gene with suggestive effects on verbal executive processing was *ROBO1*, previously implicated in verbal phonological processing (Bates et al. 2011). Whereas our SNP replication results were only nominally significant, the consistent direction of effect with the majority of previous studies boosts evidence in favour of these effects being true findings. Based on findings for other related complex traits (e.g., general cognitive ability) (Hill et al. 2018), individual SNP effects are likely to be very small (< .5% variance), so our findings align with this framework. It is widely assumed that common learning disabilities represent the low tail of normal abilities in the population, so it is unlikely that our failure to replicate previous dyslexia candidate genes is based on a different genetic aetiology of impaired and normal reading (see, Haworth and Plomin 2010).

*DYX1CA*/*DNAAF4* has a purported role in neuronal migration during cerebral cortex development, whereas *DIP2A* has been implicated in axon pathfinding and patterning in the central nervous system (http://www.genecards.org) (Tammimies et al. 2013; Tanaka et al. 2010; Wang et al. 2006; Zhang et al. 2015). Nevertheless, neuron migration and axon guidance pathways (as a whole) were not significantly associated with reading skill in our study. Given the incompleteness of GO terms and annotation bias towards genes that are well-studied (Haynes et al. 2018) we must be cautious to accept these null findings, and these pathways should be interrogated in the future as GO annotation improves, especially the quality of predicted gene function. Because these are only hypothesised biological pathways, the discovery of further genetic variants for reading (dis)ability might show that they are not the primary pathways involved in reading ability/specific learning disorder. Further, other genes might compensate for negative mutations in these pathways and effects of related brain plasticity may potentially differ by age and sex (Galaburda 2005; Galaburda et al. 2006; Poelmans et al. 2011).

*CYP19A1* encodes a member of the cytochrome P450 superfamily of enzymes, it is in the same genetic locus as *DYX1C1*, and appeared to contribute to the signal of the candidate gene-set based on its individual gene association. It has a role in testosterone to 17β-estradiol conversion, sex differentiation in the brain, and differentiation of specific brain regions in early mammalian development (Anthoni et al. 2012). Elevated levels of testosterone during perinatal development have been argued to contribute to learning disorder via lateralisation effects; oestrogen receptor beta signalling might be key in this process (Varshney and Nalvarte 2017). *CYP19A1* expression in human brain is related to expression of *DYX1C1*: 60% of variance in expression levels in nine regions of adult brain was shared (Anthoni et al. 2012). It is possible that most of the signal from our positive 14 candidate gene-set association stems from the combined associations for *CYP19A1* and *DYX1C1*, indicating the importance of the DYX1 locus, one of the most replicated quantitative trait loci for reading disability (Brkanac et al. 2007).

The present study highlights the potential importance that studying adults has for genetic discoveries in the area of developmental disorders, specifically reading impairment. If prenatal and early life developmental processes that affect the brain (and resulting reading ability) are in part genetically influenced then these genetic influences will be detected in adults provided that difficulties in reading acquisition are contiguous with later life ability. Both the phenotypic and genetic literature shows this to be true, with genetic influences on reading processes in the acquisition phases strongly correlated with reading performance later on (Byrne et al. 2009; Hart et al. 2013; Petrill et al. 2007), for example, in the teenage years no new genetic factors come into play (Wadsworth et al. 2001). Here, we are able to show that a candidate gene set of 14 genes that have individually been linked to reading disability, primarily in children and adolescents, are associated with reading ability in older Scottish people. By using adults in large-scale genotyping studies (arguably a more feasible design than in children) we are likely to speed up progress in gene discovery for dyslexia and potentially other learning disorders. It will be imperative, however, to establish what the most sensitive later life measures of early reading difficulties are given that over time individuals will develop strategies to mask their poor reading. The prospect of using nonvocal phonologically based group assessment (Wolff and Lundberg 2003) would be a way to quickly accrue large adult samples.

## Electronic supplementary material

Below is the link to the electronic supplementary material.

Supplementary material 1 (DOCX 206 KB)
