# Supplementary material for: The Influence of Dyslexia Candidate Genes on Reading Skill in Old Age
Source: Behav Genet. 2018 Jun 29;48(5):351–60. doi: 10.1007/s10519-018-9913-3 (PMC6097729; doi:10.1007/s10519-018-9913-3)
Supplement: Supplementary file 1 — Supplementary material 1 (DOCX 206 KB) [file 10519_2018_9913_MOESM1_ESM.docx]

Online Table 1. Meta-analysis association results for individual SNPs within reading disability candidate genes with reading ability measured in LBC1921 and LBC1936. Nominally significant SNP associations (P<.05) are presented in bold, although corrected significance was judged by P<.001.

| SNP ID | Chr | Gene | Effect Allele (frequency*) | Effect | SE | P-value | Significant in Previous Study |
| --- | --- | --- | --- | --- | --- | --- | --- |
| rs7523017 | 1 | *KIAA0319L* | A | 0.133 | 0.077 | 0.084 | (Couto *et al.*, 2008) |
| rs331142 | 3 | *ROBO1* | A | 0.027 | 0.047 | 0.573 | (Tran *et al.*, 2014) |
| rs333491 | 3 | *ROBO1* | A | 0.022 | 0.410 | 0.589 | (Mascheretti *et al.*, 2014) |
| rs4535189 | 3 | *ROBO1* | A | 0.017 | 0.038 | 0.653 | (Sun *et al.*, 2017) |
| rs12495133 | 3 | *ROBO1* | A | -0.005 | 0.042 | 0.900 | (Tran *et al.*, 2014) |
| rs793862 | 6 | *DCDC2* | A | 0.019 | 0.044 | 0.664 | (Scerri *et al.*, 2011, Schumacher *et al.*, 2006) |
| rs807701 | 6 | *DCDC2* | A | 0.029 | 0.041 | 0.481 | (Newbury *et al.*, 2011, Scerri *et al.*, 2011) |
| rs807724 | 6 | *DCDC2* | T | -0.020 | 0.047 | 0.669 | (Chen *et al.*, 2017, Newbury *et al.*, 2011, Scerri *et al.*, 2011) |
| rs1091047 | 6 | *DCDC2* | C | 0.032 | 0.053 | 0.551 | (Lind *et al.*, 2010) |
| rs1419228 | 6 | *DCDC2* | A | -0.068 | 0.052 | 0.189 | (Lind *et al.*, 2010) |
| rs2274305 | 6 | *DCDC2* | T | -0.029 | 0.041 | 0.486 | (Chen *et al.*, 2017, Matsson *et al.*, 2015) |
| rs4599626 | 6 | *DCDC2* | A | 0.020 | 0.044 | 0.652 | (Chen *et al.*, 2017) |
| rs6922023 | 6 | *DCDC2* | A | 0.012 | 0.053 | 0.822 | (Chen *et al.*, 2017) |
| rs6937665 | 6 | *DCDC2* | A | 0.061 | 0.053 | 0.250 | (Matsson *et al.*, 2015) |
| rs7765678 | 6 | *DCDC2* | T | -0.007 | 0.072 | 0.919 | (Lind *et al.*, 2010, Muller *et al.*, 2016) |
| rs9467075 | 6 | *DCDC2* | A | 0.033 | 0.056 | 0.556 | (Chen *et al.*, 2017, Lind *et al.*, 2010) |
| rs9467076 | 6 | *DCDC2* | T | -0.011 | 0.062 | 0.858 | (Lind *et al.*, 2010) |
| rs761100 | 6 | *KIAA0319* | A | -0.001 | 0.040 | 0.975 | (Carrion-Castillo *et al.*, 2017, Harold *et al.*, 2006, Newbury *et al.*, 2011) |
| rs2038137 | 6 | *KIAA0319* | T | -0.015 | 0.041 | 0.719 | (Carrion-Castillo *et al.*, 2017, Cope *et al.*, 2005, Harold *et al.*, 2006, Muller *et al.*, 2016) |
| rs2179515 | 6 | *KIAA0319* | T | -0.025 | 0.040 | 0.536 | (Cope *et al.*, 2005) |
| rs4504469 | 6 | *KIAA0319* | T | -0.018 | 0.039 | 0.653 | (Cope *et al.*, 2005, Harold *et al.*, 2006, Shao *et al.*, 2015, Venkatesh *et al.*, 2013) |
| rs6935076 | 6 | *KIAA0319* | T | 0.045 | 0.041 | 0.264 | (Carrion-Castillo *et al.*, 2017, Cope *et al.*, 2005, Couto *et al.*, 2010, Harold *et al.*, 2006, Muller *et al.*, 2016, Scerri *et al.*, 2011)} |
| rs9461045 | 6 | *KIAA0319* | T | -0.072 | 0.050 | 0.156 | (Scerri *et al.*, 2011, Shao *et al.*, 2015) |
| **rs759178** | **7** | ***CNTNAP2*** | **A (.50)** | **0.085** | **0.040** | **0.035** | **(Vernes *et al.*, 2008, Whitehouse *et al.*, 2011)** |
| rs851715 | 7 | *CNTNAP2* | T | -0.071 | 0.044 | 0.104 | (Vernes *et al.*, 2008) |
| **rs2538991** | **7** | ***CNTNAP2*** | **A (.50)** | **0.083** | **0.040** | **0.037** | **(Vernes *et al.*, 2008)** |
| rs2538976 | 7 | *CNTNAP2* | T | 0.073 | 0.040 | 0.068 | (Vernes *et al.*, 2008) |
| **rs2710102** | **7** | ***CNTNAP2*** | **A (.50)** | **0.085** | **0.040** | **0.034** | **(Vernes *et al.*, 2008, Whitehouse *et al.*, 2011)** |
| rs2710117 | 7 | *CNTNAP2* | A | -0.068 | 0.042 | 0.106 | (Vernes *et al.*, 2008) |
| **rs4431523** | **7** | ***CNTNAP2*** | **T (.32)** | **0.114** | **0.043** | **0.007** | **(Vernes *et al.*, 2008)** |
| rs10246256 | 7 | *CNTNAP2* | T | -0.071 | 0.043 | 0.100 | (Vernes *et al.*, 2008) |
| **rs17236239** | **7** | ***CNTNAP2*** | **A (.34)** | **0.104** | **0.042** | **0.013** | **(Carrion-Castillo *et al.*, 2017, Vernes *et al.*, 2008, Whitehouse *et al.*, 2011)** |
| rs600753 | 15 | *DYX1C1* | T | -0.035 | 0.039 | 0.370 | (Matsson *et al.*, 2015) |
| **rs3743204** | **15** | ***DYX1C1*** | **T (.23)** | **0.131** | **0.046** | **0.005** | **(Bates *et al.*, 2010)** |
| rs3743205 | 15 | *DYX1C1* | T | 0.007 | 0.106 | 0.950 | (Taipale *et al.*, 2003) |
| **rs7174102** | **15** | ***DYX1C1*** | **A (.36)** | **-0.097** | **0.041** | **0.018** | **(Paracchini *et al.*, 2011)** |
| rs8037376 | 15 | *DYX1C1* | T | 0.066 | 0.041 | 0.111 | (Paracchini *et al.*, 2011) |
| **rs8040756** | **15** | ***DYX1C1*** | **A (.15)** | **0.125** | **0.055** | **0.024** | **(Paracchini *et al.*, 2011)** |
| rs8043049 | 15 | *DYX1C1* | T | 0.078 | 0.041 | 0.054 | (Paracchini *et al.*, 2011) |
| rs17819126 | 15 | *DYX1C1* | T | 0.099 | 0.067 | 0.141 | (Bates *et al.*, 2010) |
| rs57809907 | 15 | *DYX1C1* | A | -0.030 | 0.069 | 0.664 | (Brkanac *et al.*, 2007, Dahdouh *et al.*, 2004, Scerri *et al.*, 2004, Taipale *et al.*, 2003, Wigg *et al.*, 2004) |
| rs2289105 | 15 | *CYP19A1* | T | -0.070 | 0.038 | 0.069 | (Matsson *et al.*, 2015) |
| rs4265801 | 16 | *CMIP* | T | 0.049 | 0.039 | 0.207 | (Newbury *et al.*, 2009) |
| rs6564903 | 16 | *CMIP* | T | -0.050 | 0.040 | 0.211 | (Newbury *et al.*, 2009, Scerri *et al.*, 2011) |
| rs7201632 | 16 | *CMIP* | T | 0.062 | 0.039 | 0.116 | (Newbury *et al.*, 2009) |
| rs12927866 | 16 | *CMIP* | T | -0.044 | 0.040 | 0.281 | (Scerri *et al.*, 2011) |
| rs16955705 | 16 | *CMIP* | A | 0.056 | 0.039 | 0.153 | (Newbury *et al.*, 2009, Scerri *et al.*, 2011) |
| rs2839227 | 21 | *PCNT* | A | 0.025 | 0.057 | 0.661 |  |
| rs2839232 | 21 | *PCNT* | A | 0.015 | 0.049 | 0.765 |  |
| rs2839259 | 21 | *PCNT* | T | -0.102 | 0.078 | 0.190 |  |
| rs4819241 | 21 | *PCNT* | T | -0.036 | 0.059 | 0.540 |  |
| rs762254 | 21 | *DIP2A* | A | 0.121 | 0.074 | 0.102 |  |
| **rs1892692** | **21** | ***DIP2A*** | **A (.41)** | **-0.099** | **0.039** | **0.012** |  |
| rs2070435 | 21 | *DIP2A* | A | -0.073 | 0.041 | 0.074 |  |
| rs2255526 | 21 | *DIP2A* | A | -0.003 | 0.045 | 0.940 |  |
| rs2839282 | 21 | *DIP2A* | C | 0.127 | 0.076 | 0.096 |  |
| rs2839299 | 21 | *DIP2A* | T | 0.067 | 0.042 | 0.106 |  |
| rs2839308 | 21 | *DIP2A* | A | 0.033 | 0.047 | 0.485 |  |
| rs8132320 | 21 | *DIP2A* | A | -0.019 | 0.050 | 0.705 |  |
| rs16979358 | 21 | *DIP2A* | T | 0.061 | 0.077 | 0.426 |  |
| rs17302525 | 21 | *DIP2A* | A | -0.113 | 0.082 | 0.170 |  |
| rs9722 | 21 | *S100B* | A | -0.052 | 0.069 | 0.452 | (Matsson *et al.*, 2015) |

Note: Alleles are coded on the positive strand.

* In the larger cohort, LBC1936.

Online Table 2. Meta-analysis association results for individual SNPs within reading disability candidate genes with verbal executive processing measured in LBC1921 and LBC1936. Nominally significant SNP associations are presented in bold.

| SNP ID | Chromosome | Gene | Effect Allele (frequency*) | Effect | SE | P-value |
| --- | --- | --- | --- | --- | --- | --- |
| rs7523017 | 1 | *KIAA0319L* | A | 0.094 | 0.797 | 0.906 |
| rs331142 | 3 | *ROBO1* | A | -0.896 | 0.488 | 0.066 |
| **rs333491** | **3** | ***ROBO1*** | **A (.57)** | **0.827** | **0.418** | **0.048** |
| rs4535189 | 3 | *ROBO1* | A | 0.357 | 0.390 | 0.361 |
| **rs12495133** | **3** | ***ROBO1*** | **A (.36)** | **-1.159** | **0.42** | **0.006** |
| rs793862 | 6 | *DCDC2* | A | -0.374 | 0.454 | 0.410 |
| rs807701 | 6 | *DCDC2* | A | -0.077 | 0.420 | 0.855 |
| rs807724 | 6 | *DCDC2* | T | 0.252 | 0.479 | 0.599 |
| rs1091047 | 6 | *DCDC2* | C | -0.651 | 0.547 | 0.234 |
| rs1419228 | 6 | *DCDC2* | A | -0.312 | 0.535 | 0.560 |
| rs2274305 | 6 | *DCDC2* | T | 0.074 | 0.422 | 0.860 |
| rs4599626 | 6 | *DCDC2* | A | 0.704 | 0.447 | 0.115 |
| rs6922023 | 6 | *DCDC2* | A | 0.348 | 0.541 | 0.520 |
| rs6937665 | 6 | *DCDC2* | A | 0.468 | 0.540 | 0.386 |
| rs7765678 | 6 | *DCDC2* | T | -0.659 | 0.721 | 0.361 |
| rs9467075 | 6 | *DCDC2* | A | -0.107 | 0.577 | 0.852 |
| rs9467076 | 6 | *DCDC2* | T | 0.508 | 0.637 | 0.425 |
| rs761100 | 6 | *KIAA0319* | A | -0.609 | 0.408 | 0.136 |
| rs2038137 | 6 | *KIAA0319* | T | -0.672 | 0.417 | 0.107 |
| rs2179515 | 6 | *KIAA0319* | T | -0.648 | 0.412 | 0.116 |
| rs4504469 | 6 | *KIAA0319* | T | -0.516 | 0.404 | 0.202 |
| rs6935076 | 6 | *KIAA0319* | T | 0.230 | 0.413 | 0.577 |
| rs9461045 | 6 | *KIAA0319* | T | 0.507 | 0.519 | 0.329 |
| rs759178 | 7 | *CNTNAP2* | A | 0.081 | 0.410 | 0.843 |
| rs851715 | 7 | *CNTNAP2* | T | -0.096 | 0.446 | 0.830 |
| rs2710102 | 7 | *CNTNAP2* | A | 0.084 | 0.410 | 0.837 |
| rs2710117 | 7 | *CNTNAP2* | A | 0.087 | 0.432 | 0.840 |
| rs2538991 | 7 | *CNTNAP2* | A | 0.087 | 0.409 | 0.831 |
| rs2538976 | 7 | *CNTNAP2* | T | 0.009 | 0.407 | 0.983 |
| rs4431523 | 7 | *CNTNAP2* | T | 0.443 | 0.436 | 0.310 |
| rs10246256 | 7 | *CNTNAP2* | T | -0.121 | 0.438 | 0.783 |
| rs17236239 | 7 | *CNTNAP2* | A | -0.068 | 0.426 | 0.873 |
| rs600753 | 15 | *DYX1C1* | T | -0.439 | 0.401 | 0.274 |
| rs3743204 | 15 | *DYX1C1* | T | 0.686 | 0.475 | 0.149 |
| rs3743205 | 15 | *DYX1C1* | T | 0.411 | 1.121 | 0.714 |
| rs7174102 | 15 | *DYX1C1* | A | -0.275 | 0.421 | 0.514 |
| rs8037376 | 15 | *DYX1C1* | T | 0.175 | 0.425 | 0.681 |
| rs8040756 | 15 | *DYX1C1* | A | 0.398 | 0.563 | 0.479 |
| rs8043049 | 15 | *DYX1C1* | T | 0.080 | 0.419 | 0.849 |
| rs17819126 | 15 | *DYX1C1* | T | 0.773 | 0.698 | 0.268 |
| rs57809907 | 15 | *DYX1C1* | A | -0.295 | 0.699 | 0.673 |
| rs2289105 | 15 | *CYP19A1* | T | 0.019 | 0.394 | 0.962 |
| rs3935802 | 16 | *CMIP* | C | -0.396 | 0.408 | 0.331 |
| rs4265801 | 16 | *CMIP* | T | 0.437 | 0.398 | 0.272 |
| rs6564903 | 16 | *CMIP* | T | -0.496 | 0.410 | 0.227 |
| rs7201632 | 16 | *CMIP* | T | 0.473 | 0.404 | 0.241 |
| rs12927866 | 16 | *CMIP* | T | -0.347 | 0.414 | 0.402 |
| rs16955705 | 16 | *CMIP* | A | 0.381 | 0.405 | 0.347 |
| rs2839227 | 21 | *PCNT* | A | 0.469 | 0.593 | 0.429 |
| rs2839232 | 21 | *PCNT* | A | -0.577 | 0.510 | 0.258 |
| rs2839259 | 21 | *PCNT* | T | 0.213 | 0.790 | 0.787 |
| rs4819241 | 21 | *PCNT* | T | -0.735 | 0.617 | 0.233 |
| rs762254 | 21 | *DIP2A* | A | -0.149 | 0.747 | 0.842 |
| rs1892692 | 21 | *DIP2A* | A | -0.018 | 0.401 | 0.965 |
| rs2070435 | 21 | *DIP2A* | A | 0.305 | 0.423 | 0.471 |
| rs2255526 | 21 | *DIP2A* | A | 0.530 | 0.463 | 0.253 |
| rs2839282 | 21 | *DIP2A* | C | 0.754 | 0.750 | 0.315 |
| rs2839299 | 21 | *DIP2A* | T | 0.501 | 0.429 | 0.243 |
| **rs2839308** | **21** | ***DIP2A*** | **A (.24)** | **1.131** | **0.481** | **0.019** |
| **rs8132320** | **21** | ***DIP2A*** | **A (.20)** | **-1.135** | **0.517** | **0.028** |
| rs16979358 | 21 | *DIP2A* | T | -0.320 | 0.788 | 0.684 |
| rs17302525 | 21 | *DIP2A* | A | 0.391 | 0.844 | 0.644 |
| rs9722 | 21 | *S100B* | A | 1.073 | 0.689 | 0.119 |

Note: Alleles are coded on the positive strand.

* In the larger cohort, LBC1936

Online Figure 1. QQ Plot for dyslexia candidate gene SNP associations (N = 9,225 SNPs) with Verbal Executive Processing.


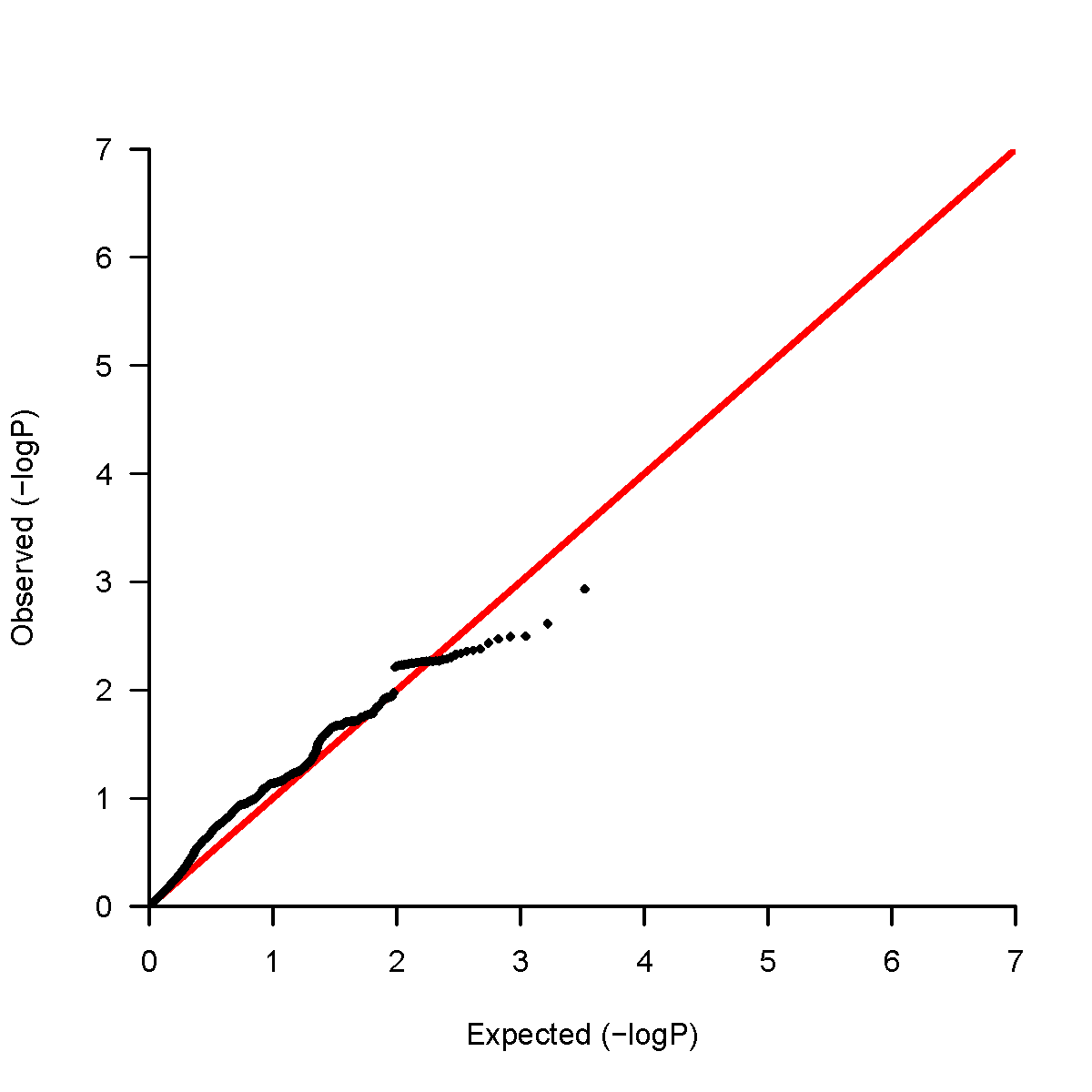


**References**

Bates, T.C., Lind, P.A., Luciano, M., Montgomery, G.W., Martin, N.G. & Wright, M.J. (2010) Dyslexia and DYX1C1: deficits in reading and spelling associated with a missense mutation. *Mol Psychiatr,* **15,** 1190-1196.

Brkanac, Z., Chapman, N.H., Matsushita, M.M., Chun, L., Nielsen, K., Cochrane, E., Berninger, V.W., Wijsman, E.M. & Raskind, W.H. (2007) Evaluation of candidate genes for DYX1 and DYX2 in families with dyslexia. *Am J Med Genet B,* **144B,** 556-560.

Carrion-Castillo, A., Maassen, B., Franke, B., Heister, A., Naber, M., van der Leij, A., Francks, C. & Fisher, S.E. (2017) Association analysis of dyslexia candidate genes in a Dutch longitudinal sample. *Eur J Hum Genet,* **25,** 452-460.

Chen, Y., Zhao, H., Zhang, Y.-x. & Zuo, P.-x. (2017) DCDC2 gene polymorphisms are associated with developmental dyslexia in Chinese Uyghur children. *Neural Regeneration Research,* **12,** 259-266.

Cope, N., Harold, D., Hill, G., Moskvina, V., Stevenson, J., Holmans, P., Owen, M.J., O’Donovan, M.C. & Williams, J. (2005) Strong evidence that KIAA0319 on chromosome 6p is a susceptibility gene for developmental dyslexia. *The American Journal of Human Genetics,* **76,** 581-591.

Couto, J.M., Gomez, L., Wigg, K., Cate-Carter, T., Archibald, J., Anderson, B., Tannock, R., Kerr, E.N., Lovett, M.W., Humphries, T. & Barr, C.L. (2008) The KIAA0319-Like (KIAA0319L) Gene on Chromosome 1p34 as a Candidate for Reading Disabilities. *J Neurogenet,* **22,** 295-313.

Couto, J.M., Livne-Bar, I., Huang, K., Xu, Z., Cate-Carter, T., Feng, Y., Wigg, K., Humphries, T., Tannock, R., Kerr, E.N., Lovett, M.W., Bremner, R. & Barr, C.L. (2010) Association of reading disabilities with regions marked by acetylated H3 histones in KIAA0319. *American journal of medical genetics. Part B, Neuropsychiatric genetics : the official publication of the International Society of Psychiatric Genetics,* **153B,** 447-462.

Dahdouh, F., Schumacher, J., Koenig, I.R., Lindgren, C., Peyrard-Janvid, M., Anthoni, H., Zucchelli, M., Grimm, T., Warnke, A., Roth, E., Cichon, S., Remschmidt, H., Propping, P., Ziegler, A., Kere, J., Schulte-Korne, G. & Nothen, M.M. (2004) Examination of a possible influence of genetic variation at the DYX1C1 locus in the development of dyslexia. *Am J Med Genet B,* **130B,** 106-106.

Harold, D., Paracchini, S., Scerri, T., Dennis, M., Cope, N., Hill, G., Moskvina, V., Walter, J., Richardson, A.J., Owen, M.J., Stein, J.F., D Green, E., O'Donovan, M.C., Williams, J. & Monaco, A.P. (2006) Further evidence that the KIAA0319 gene confers susceptibility to developmental dyslexia. *Mol Psychiatr,* **11,** 1085-1091.

Lind, P.A., Luciano, M., Wright, M.J., Montgomery, G.W., Martin, N.G. & Bates, T.C. (2010) Dyslexia and DCDC2: normal variation in reading and spelling is associated with DCDC2 polymorphisms in an Australian population sample. *Eur J Hum Genet,* **18,** 668-673.

Mascheretti, S., Riva, V., Giorda, R., Beri, S., Lanzoni, L.F.E., Cellino, M.R. & Marino, C. (2014) KIAA0319 and ROBO1: evidence on association with reading and pleiotropic effects on language and mathematics abilities in developmental dyslexia. *J Hum Genet,* **59,** 189-197.

Matsson, H., Huss, M., Persson, H., Einarsdottir, E., Tiraboschi, E., Nopola-Hemmi, J., Schumacher, J., Neuhoff, N., Warnke, A., Lyytinen, H., Schulte-Korne, G., Nothen, M.M., Leppanen, P.H., Peyrard-Janvid, M. & Kere, J. (2015) Polymorphisms in DCDC2 and S100B associate with developmental dyslexia. *J Hum Genet,* **60,** 399-401.

Muller, B., Wilcke, A., Czepezauer, I., Ahnert, P., Boltze, J., Kirsten, H. & consortium, L. (2016) Association, characterisation and meta-analysis of SNPs linked to general reading ability in a German dyslexia case-control cohort. *Scientific reports,* **6,** 27901.

Newbury, D.F., Paracchini, S., Scerri, T.S., Winchester, L., Addis, L., Richardson, A.J., Walter, J., Stein, J.F., Talcott, J.B. & Monaco, A.P. (2011) Investigation of Dyslexia and SLI Risk Variants in Reading- and Language-Impaired Subjects. *Behav Genet,* **41,** 90-104.

Newbury, D.F., Winchester, L., Addis, L., Paracchini, S., Buckingham, L.L., Clark, A., Cohen, W., Cowie, H., Dworzynski, K., Everitt, A., Goodyer, I.M., Hennessy, E., Kindley, A.D., Miller, L.L., Nasir, J., O'Hare, A., Shaw, D., Simkin, Z., Simonoff, E., Slonims, V., Watson, J., Ragoussis, J., Fisher, S.E., Seckl, J.R., Helms, P.J., Bolton, P.F., Pickles, A., Conti-Ramsden, G., Baird, G., Bishop, D.V.M. & Monaco, A.P. (2009) CMIP and ATP2C2 Modulate Phonological Short-Term Memory in Language Impairment. *Am J Hum Genet,* **85,** 264-272.

Paracchini, S., Ang, Q.W., Stanley, F.J., Monaco, A.P., Pennell, C.E. & Whitehouse, A.J. (2011) Analysis of dyslexia candidate genes in the Raine cohort representing the general Australian population. *Genes, brain, and behavior,* **10,** 158-165.

Scerri, T.S., Fisher, S.E., Francks, C., MacPhie, I.L., Paracchini, S., Richardson, A.J., Stein, J.F. & Monaco, A.P. (2004) Putative functional alleles of DYX1C1 are not associated with dyslexia susceptibility in a large sample of sibling pairs from the UK. *J Med Genet,* **41,** 853-857.

Scerri, T.S., Morris, A.P., Buckingham, L.L., Newbury, D.F., Miller, L.L., Monaco, A.P., Bishop, D.V.M. & Paracchini, S. (2011) DCDC2, KIAA0319 and CMIP Are Associated with Reading-Related Traits. *Biol Psychiat,* **70,** 237-245.

Schumacher, J., Anthoni, H., Dahdouh, F., König, I.R., Hillmer, A.M., Kluck, N., Manthey, M., Plume, E., Warnke, A. & Remschmidt, H. (2006) Strong genetic evidence of DCDC2 as a susceptibility gene for dyslexia. *The American Journal of Human Genetics,* **78,** 52-62.

Shao, S., Kong, R., Zou, L., Zhong, R., Lou, J., Zhou, J., Guo, S., Wang, J., Zhang, X., Zhang, J. & Song, R. (2015) The Roles of Genes in the Neuronal Migration and Neurite Outgrowth Network in Developmental Dyslexia: Single- and Multiple-Risk Genetic Variants. *Mol Neurobiol*.

Sun, X., Song, S., Liang, X., Xie, Y., Zhao, C., Zhang, Y., Shu, H. & Gong, G. (2017) ROBO1 polymorphisms, callosal connectivity, and reading skills. *Human brain mapping,* **38,** 2616-2626.

Taipale, M., Kaminen, N., Nopola-Hemmi, J., Haltia, T., Myllyluoma, B., Lyytinen, H., Muller, K., Kaaranen, M., Lindsberg, P.J., Hannula-Jouppi, K. & Kere, J. (2003) A candidate gene for developmental dyslexia encodes a nuclear tetratricopeptide repeat domain protein dynamically regulated in brain. *P Natl Acad Sci USA,* **100,** 11553-11558.

Tran, C., Wigg, K.G., Zhang, K., Cate-Carter, T.D., Kerr, E., Field, L.L., Kaplan, B.J., Lovett, M.W. & Barr, C.L. (2014) Association of the ROBO1 gene with reading disabilities in a family- based analysis. *Genes Brain Behav,* **13,** 430-438.

Venkatesh, S.K., Siddaiah, A., Padakannaya, P. & Ramachandra, N.B. (2013) Analysis of genetic variants of dyslexia candidate genes KIAA0319 and DCDC2 in Indian population. *J Hum Genet,* **58,** 531-538.

Vernes, S.C., Newbury, D.F., Abrahams, B.S., Winchester, L., Nicod, J., Groszer, M., Alarcon, M., Oliver, P.L., Davies, K.E., Geschwind, D.H., Monaco, A.P. & Fisher, S.E. (2008) A Functional Genetic Link between Distinct Developmental Language Disorders. *New Engl J Med,* **359,** 2337-2345.

Whitehouse, A.J.O., Bishop, D.V.M., Ang, Q.W., Pennell, C.E. & Fisher, S.E. (2011) CNTNAP2 variants affect early language development in the general population. *Genes, Brain and Behavior,* **10,** 451-456.

Wigg, K.G., Couto, J.M., Feng, Y., Anderson, B., Cate-Carter, T.D., Macciardi, F., Tannock, R., Lovett, M.W., Humphries, T.W. & Barr, C.L. (2004) Support for EKN1 as the susceptibility locus for dyslexia on 15q21. *Mol Psychiatry,* **9,** 1111-1121.
